# Prevalence of Rotavirus Genogroup A and Norovirus Genogroup II in Bassaseachic Falls National Park Surface Waters in Chihuahua, Mexico

**DOI:** 10.3390/ijerph14050482

**Published:** 2017-05-05

**Authors:** Ma. Carmen E. Delgado-Gardea, Patricia Tamez-Guerra, Ricardo Gomez-Flores, Aurora Mendieta-Mendoza, Francisco Javier Zavala-Díaz de la Serna, Juan Francisco Contreras-Cordero, Gilberto Erosa-de la Vega, María Concepción Pérez-Recoder, Blanca Sánchez-Ramírez, Carmen González-Horta, Rocío Infante-Ramírez

**Affiliations:** 1 Laboratorio de Biotecnología, Facultad de Ciencias Químicas, Universidad Autónoma de Chihuahua, Circuito Nuevo Campus Universitario No.1, Chihuahua 31125, Mexico; carmen_060@hotmail.com (M.C.E.D.-G.); mema_bora@hotmail.com (A.M.-M.); fzavala@uach.mx (F.J.Z.-D.d.l.S.); gerosa@uach.mx (G.E.-d.l.V.); bsanche@uach.mx (B.S.-R.); carmengonzalez@uach (C.G.-H.); 2 Departamento de Microbiología e Inmunología, Facultad de Ciencias Biológicas, Universidad Autónoma de Nuevo León, Ave. Universidad s/n, San Nicolás de los Garza 66455, Mexico; patamez@hotmail.com (P.T.-G.); rgomez60@hotmail.com (R.G.-F.); contrerasjfco@gmail.com (J.F.C.-C.); 3 Comisión Nacional de Áreas Naturales Protegidas, Dirección Regional Norte y Sierra Madre Occidental, Parque Nacional Cascada de Bassaseachic, Ocampo, Chihuahua 31203, Mexico; crecoder@conanp.gob.mx

**Keywords:** norovirus, rotavirus, surface water, VIRADEL, virus detection, environment

## Abstract

In areas lacking potable water treatment, drinking contaminated water may represent a public health threat. In addition to enteropathogenic bacteria and parasites, fecal contamination in water environments is associated with the transmission of enteric viruses and other causal agents of infectious disease. Rotavirus and norovirus are the main enteric viral agents responsible for diarrheic outbreaks. The aim of the present study was to detect seasonal variation of rotavirus and norovirus in the surface water at Bassaseachic Falls National Park during 2013. Rivers and streams within and nearby this park were sampled once in each season during 2013. Viral concentration was carried out by a handmade filtration equipment, using a commercial electropositive membrane coupled with the virus absortion elution technique (VIRADEL©). Detection of rotavirus and norovirus was performed by SYBR Green reverse transcription-real time polymerase chain reaction (SYBR GREEN© RT-qPCR) analyses. Norovirus genogroup II was detected in samples collected in June and October 2013. In the case of rotavirus, genogroup A was detected in March and June. The presence of rotavirus and norovirus was related to viral acute diarrhea in children less than five years of age, who were inhabiting the sampled areas. This may indicates that the contaminated water was potentially a risk factor for regional diarrheic outbreaks.

## 1. Introduction

Viral gastroenteritis outbreaks are associated with water contamination due to wastewater containing the viral particles, which is discharged in surface waters and rivers. Infectious agents can be spread in this way, particularly during periods of low flow [1,2]. Although the number of viral particles in the surface water is very low, ten viral particles are sufficient for a healthy individual to be infected [3,4].

Bassaseachic Falls National Park is located in the western side of the state of Chihuahua, Mexico, in the Sierra Madre Occidental. Basaseachi Waterfall is the main flowing part of the Basaseachi and Durazno micro-basins. This park is a natural, protected area that is considered a unique ecosystem, with endemic flora and wildlife species. Environmental conditions favor the development of a forest ecosystem in the highest areas, whereas in lower areas, such as “Barranca de Candameña”, the ecosystem is that of jungle vegetation. Bassaseachic Falls National park has peculiar climatic characteristics as a result of the latitude and altitude, with temperatures ranging from 26 °C to 16 °C. There is frosty weather from October to May, while there is snowy weather from November to March [5].

It is usually difficult to detect viruses in water samples because of their very low concentration and the presence of inhibitors, such as humic and fulvic acid, polysaccharides, bacterial debris, nucleases and metal ions that make it difficult to extract RNA. For this reason, the detection of viruses in water requires specific methods involving viral particle concentration, such as ultrafiltration. Large water volumes (100–1000 L) usually need to be processed. Ultrafiltration techniques with electropositive commercial membranes, coupled with a SYBR Green reverse transcription-real time polymerase chain reaction (RT-qPCR) assay are inexpensive and useful to amplify genes for detecting low virus concentrations [6,7,8]. Using this method, in recent years, more than 100 types of viruses that can be transmitted to humans have been reported in contaminated waters, such as adenovirus (AdV), rotavirus (RV), norovirus (NV) and hepatitis virus (HepV) [9]. RV and NV are the major etiological agents responsible for the known cases of viral gastroenteritis worldwide [10]. The aim of the present study was to detect RV and NV as well as their concentration variations in the surface water of Bassaseachic Falls National Park during the seasons in 2013.

## 2. Experimental Section

### 2.1. Collection of Samples

**Figure 1:**
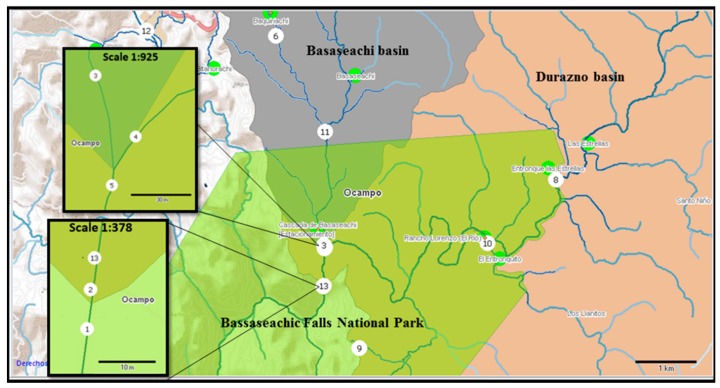
Bassaseachic Falls National Park geographical area. The numbered circles indicate the sampling sites. Source: INEGI digital map, Cartographic Package 2010; Scale 1:41062. Samples 5 (28°10′47.45″ N, 108°12′45.49″ W) and 6 (28°12′40.80″ N, 108°13′20.49″ W) during June were missing, while sample 13 during March was also missing (28_10127.36″ N, 108_12144.97″ W). See Supplementary Materials Table S1 for sampling site names.

The sites sampled, as previously determined by the Mexican governmental agency “Statistics and Geography National Institute” (Instituto Nacional de Estadística y Geografía—INEGI), are shown in Figure 1.

In Bassaseachic Falls National Park, we selected 13 sampling points located near rural settlements which may drain their sewage into the surface waters. In order to detect enteric viruses in the water, 1 L samples were collected in UV-light sterile polypropylene bottles [11] during March (spring conditions), June (drought conditions), October (raining conditions) and December (winter conditions) in 2013. Sample 13 was not obtained in March due to poor safety conditions, while samples 5 and 6 were not obtained in June because of heavy drought. There was a total of 49 samples (Supplementary Materials Table S1). Samples 1, 2 and 13 were collected in Basaseachi Falls, sample 3 was from Durazno River, sample 4 was from Basaseachi River, while sample 5 was collected from the river connecting both rivers, also known as “Y River.” Sample 6 was from the “Baquirichi” stream, sample 7 from the water supply stream for Basaseachi City, and sample 8 was from the “Las Estrellas” stream. Sample 9 was obtained from the visitor center of Bassaseachic Falls National Park, sample 10 was from the “Betorachi” stream, sample 11 was from the creek next to the oxidation pond and sample 12 from the “Cahuisori” stream. The presence of viruses was determined by RT-PCR, while RT-qPCR SYBR Green© was used for virus quantification [6,7]. For further analyses, all monthly collected samples were pooled to increase the analyzed water volume, in order to avoid false negative results.

### 2.2. Virus Adsorption-Elution Technique (VIRADEL)

All samples were stored with ice inside the sterile propylene bottles until use. For the adsorption of viruses, water samples were filtered with a handmade filtration equipment through an electropositive commercial cellulose membrane, which had a pore size of 0.47 μm (VCM-47mm^®^, Scientific Methods, Inc., Granger, IN, USA). Filtration was conducted using a vacuum pump system with a flow rate of 1.072 L min^−1^ (Toolcraft^®^, Dayton, OH, USA). For the recovery of viral particles from the electropositive membrane, membranes were eluted by organic flocculation. For this, 15 mL of eluent solution containing beef extract broth 1%, pH 9 ± 0.1 (Sigma-Aldrich, St. Louis, MO, USA), 0.5 M glycine (JT Baker, S.A. de C.V., Ecatepec, Estado de México, México) and 1% tween 80 solution (JT Baker) were added to the electropositive membrane in a sterile Petri dish and mixed for 30 min. Following this, pH was adjusted to be 3.5 ± 0.1 using 1 M of HCl. A 3% solution of the beef extract powder was then prepared and centrifuged for 30 min at 2600 *g* for the second step, which involved re-concentration of the viral particles. After this, the precipitate was dissolved in 1 mL of phosphate buffer saline (PBS) (pH 7.0–7.05) as part of the viral suspension, and stored at −20 °C until RNA extraction was commenced [12]. This technique has a virus concentration detection efficiency of 10 copies.

### 2.3. Viral RNA Extraction Using Trizol^®^ Method

Using 300 µL of the viral suspension, total RNA extraction was performed by the Trizol method, according to Chomczynski et al. [13].

### 2.4. Reverse Transcription (RT) for RV

RNA extracted was transcribed into complementary DNA (cDNA) using the reverse primer CON 2 (Table 1), according to Gentsch et al. [14] (Supplementary Materials Table S2). RV virus cDNA was then amplified during two RT-PCR steps using the M-MLV enzyme (PROMEGA, Fitchburg, WI, USA). The amplified product was then analyzed on 1% agarose gel (Bioline, Tauton, MA, USA).

### 2.5. Polymerase Chain Reaction (PCR) for RV

To determine the genogroup, an amplicon of the virus protein 4 (VP4), with an expected size of 345 bp, was used (Supplementary Materials Table S3).

### 2.6. RT for NV Detection

RNA extracted from the viral suspension was transcribed into complementary DNA (cDNA), using the reverse primer JV13 (Table 2), according to Thorven et al. [15] (Supplementary Materials Table S4). This product was then amplified with one RT-PCR step using the M-MLV enzyme (PROMEGA, Fitchburg, WI, USA). The amplified product was then analyzed on 1% agarose gel (Bioline, Tauton, MA, USA).

### 2.7. PCR for NV Detection

To determine the genogroup, the selected amplicon for NV detection was the RNA-dependent RNA polymerase (RdPd), which is a fragment with an expected size of 326 bp (Supplementary Materials Table S5).

### 2.8. Real Time PCR Amplification (qPCR)

The qPCR was performed using a commercial qPCR Master Kit Mix Brilliant III Ultra-Fast SYBR^®^ Green qPCR in a Stratagene 3005p thermocycler (Agilent Technologies, Santa Clara, CA, USA), according to the manufacturer’s instructions. RNA sample analysis for virus quantification was also run in duplicates using the cDNA standard. NV protocols were performed according to Thorven et al. [15] and Scipioni et al. [2]. RV detection was performed as reported by Gentsch et al. [14] and Kottaridi et al. [16] (Supplementary Materials Tables S6 and S7).

The thermocycler program for RV quantification was the following: 1 cycle at 95 °C for 3 min, 45 cycles at 95 °C for 30 s, 53 °C for 20 s and 72 °C for 30 s. The thermocycler program for the dissociation curve was 1 cycle at 95 °C for 60 s, 53 °C for 30 s and 95 °C for 30 s.

The thermocycler program for NV quantification was 1 cycle at 95 °C for 3 min in addition to 40 cycles at 95 °C for 10 s, 48 °C for 20 s and 60 °C for 42 s. The thermocycler program for the dissociation curve was 1 cycle at 95 °C for 60 s, 48 °C for 30 s and 95 °C for 30 s.

### 2.9. Construction of cDNA Standard

The PCR product of RV and NV from RNA extracted from feces was purified with the DNA Clean & Concentrator™-5 (Zymoresearch, Irvine, CA, USA) kit and cloned in plasmid (pGEM-T vector system I, PROMEGA, Fitchburg, WI, USA). The colonies were placed into a broth and cultured for 24 h at 37 °C in Luria-Bertani (LB) medium containing 100 µg/mL of ampicillin. Following this, the DNA was extracted, analyzed with electrophoresis and linearized by the digestion enzyme *NcoI* (R0193S) (New England Biolabs, Ipswich, MA, USA). Nanodrop 2000p equipment (Thermo Fisher Scientific Inc., Boston, MA, USA) was used to determine DNA concentration (ng/µL).

### 2.10. Standard Curve

Plasmid DNA was diluted by seven 10-fold dilutions (10^8^ to 10^2^ copies/reaction). Each sample analysis was performed in duplicate. Threshold (C_t_) values were set up with MxPRO QPCR software for Mx3000P QPCR Systems using the second derived maximum method. PCR efficiency was calculated by two methods. The first used the MxPRO QPCR software while the second method was performed according to the following equation: % Efficiency = [10^(−1/slope)^ − 1] × 100 [17]. To determine the virus quantity (copies/reaction), the following equation was used [18]:

Copies/Reaction = 6.022 × 10^23^ mol × [C] / molecular weight × 6.58 × 10^2^ g/mol × 1 × 10^9^

where [C] = linearized plasmid concentration (ng/µL).

### 2.11. Quality Control

The positive control used was the combination of 1 mL of NV positive feces added to 1 L of water with 1 mL of RV positive feces from the coproteca Facultad de Ciencias Químicas of the Universidad Autónoma de Chihuahua (FCQ-UACH) added to 1 L of water. The negative control used was 2 L of tap water. Both controls were run at the same time as the samples.

### 2.12. Sequencing

The PCR products were sent for sequence analysis (Macrogen, Korea). The nucleotide sequence analysis was performed using CLUSTALW/BioEdit sequence alignment Version 7.0. The phylogenetic tree was performed with the MEGA version 6.0 software with 1000 bootstrapped data, set with the neighbor joining method. The sequences were submitted to the GenBank database. Access numbers submitted to GenBank were assigned as GenBank grp 5981238 (registration in process).

### 2.13. Limits of Quantification

Samples, which did not amplify for the tested viruses or detect a number above the quantification limit (LOQ) in qPCR reaction replicates (i.e., amplified only one out of two samples), were reported as “not detected” (ND).

## 3. Results

### 3.1. Norovirus RT-PCR Optimization

The standard curve for NV was done in duplicate (10^8^ to 10^2^ copies/reaction). Different annealing temperatures (45 to 48 °C) and final concentrations of primer (0.2, 0.3 and 0.4 µM) were used for the evaluation. Based on the obtained data, the final concentration of primers was 0.2 µM, with an annealing temperature around 48 °C. The correlation coefficient was r^2^ > 0.97, with a slope of −3.229 (Supplementary Materials Figure S1). PCR efficiency calculated by the MxPRO qPCR software usually ranged from 89% to 104%. NV was detected during the months of June and October. The highest detection was 5.27 × 10^2^ copies/L in the samples in October, whereas the lowest detection was 1.00 × 10^2^ copies/L during the month of June (Table 2).

### 3.2. Rotavirus RT-PCR Optimization

The standard curve for RV was done in duplicate (10^8^ to 10^2^ copies/reaction). Different annealing temperatures (42 to 53 °C) and final concentrations of primer (0.4, 0.5 and 0.6 µM) were evaluated. The conditions for this test were 52 °C for the annealing temperature and 0.5 µM for the final concentration of primers. The correlation coefficient was r^2^ > 0.99, with a slope of −3.332 (Supplementary Materials Figure S2). PCR efficiency usually ranged from 90% to 100%. Amplification results demonstrated the presence of RV and NV genes among the analyzed samples.

RV was mainly detected during March and June, with the highest detection reaching 180 copies/reaction during June, whereas the lowest detection was 98 copies/reaction in the samples taken in March (Table 3).

### 3.3. Phylogenetic Sequence Analysis

**Figure 2:**
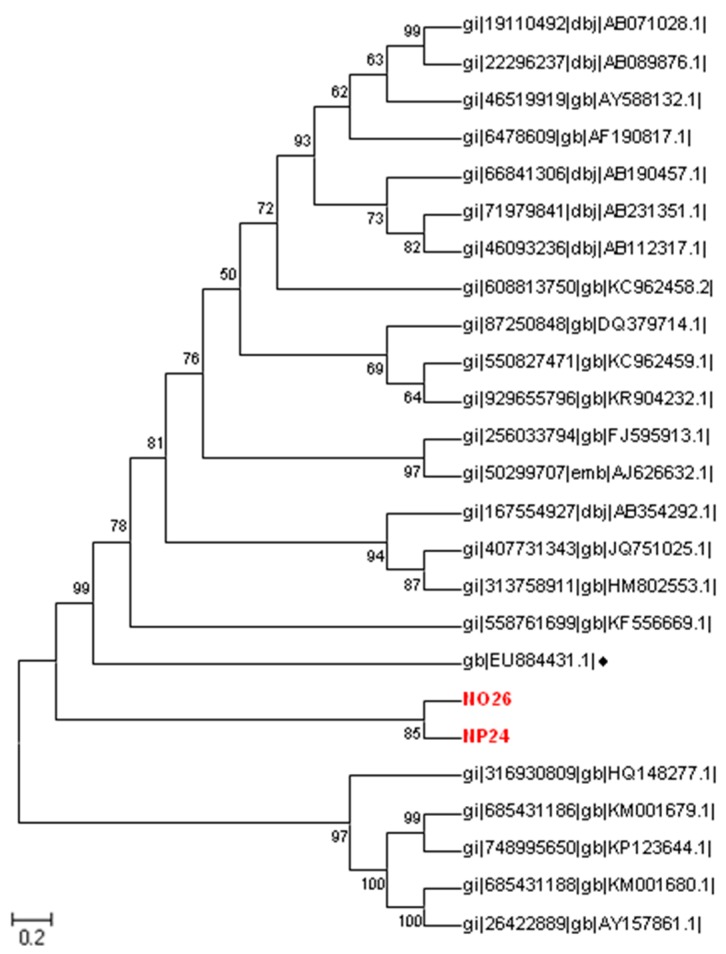
Evolution of the relationship of a region of the norovirus RdPd gene by a phylogenetic tree constructed with different strains around the world. This was inferred by neighbor joining. Our sequences NO26 and NP24 are highlighted, and the sequence EU884431 is marked with the ♦ symbol. The evolutionary distances were computed using the Kimura 2 method with a G value of 1.73. The bootstrap consensus tree inferred from 1000 replicates was taken to represent the evolutionary history of the analyzed taxa. There was a total of 160 positions in the final dataset. Evolutionary analyses were conducted using the software MEGA6.

The RV and NV positive samples were sequenced and analyzed to determine differences with those reported worldwide. Phylogenetic trees were produced for both viruses, using neighbor joining with the Kimura method and a G value of 1.73. The sequence names were NO26 for October and NP24 for June, with these sequences being compared with previous sequences reported in the GenBank. Those sequences were chosen for their relationship with the RdPd gene, as they had a similarity (about 80% identity) to a strain from Japan, the USA and Italy. The result confirms that the detected NV in both cases belonged to genogroup II. This genogroup is distributed worldwide and is associated with diarrheic outbreaks [6]. Nevertheless, it differs from the previously reported Mexican NV sequence (EU884431) (Figure 2), thus indicating the need of genotype studies to obtain accurate phenotype information.

**Figure 3:**
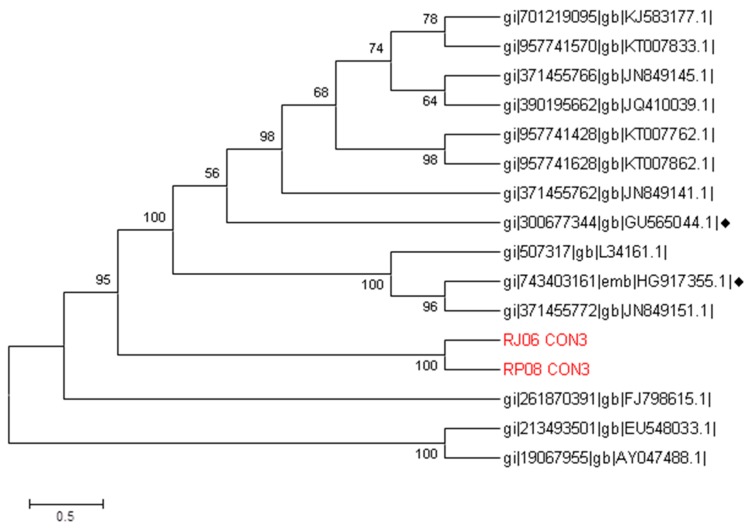
Phylogenetic tree constructed with a region of the four genes from rotavirus with different strains around the world. This was inferred by neighbor joining. Our sequences RP08 and RJ06 are highlighted along with the vaccine sequences, which are marked by the ♦ symbol. The evolutionary distances were computed using the Kimura 2 method with G value of 1.73. The bootstrap consensus tree inferred from 1000 replicates is taken to represent the evolutionary history of the analyzed taxa. There was a total of 490 positions in the final dataset. Evolutionary analyses were conducted using the software MEGA6.

In addition, the RV sequence names were RP08 for March and RJ06 for June. Their characterization was compared with the sequences of the fragment VP4 from the GenBank, which found they had similarities with strains found in the USA and Thailand in addition to the vaccine sequences, HG917355 and GU565044. Our results showed that RV belongs to the P[8] genotype (Figure 3).

## 4. Discussion

With emergency viral outbreaks associated with the transmission of waterborne pathogens, it is necessary to develop a rapid and simple method to detect norovirus and rotavirus. The detection of waterborne viruses is important in public health, especially in locations where potable water is scarce. Rotavirus and norovirus have commonly been found in aquatic environments. Due to its nature, this contamination is frequently related to biological waste disposals [19,20]. Similarly, collected data from waste water and river water during the coldest months in Japan showed NV ranges between 1.6 × 10^2^ and 8.0 × 10^3^ copies/L [21]. Recently, the use of real time PCR (qPCR) as a detection tool has been well accepted, since it is an accurate, quantitative and quick molecular biology analysis method. Before qPCR, reverse transcription PCR (RT-PCR) was the selected method to detect the presence or absence of RV and NV. Gentsch et al. [14] used this method to amplify a segment of gene 4, which codes for the outer capsid virus protein 4 (VP4) from rotavirus A, whereas Thorven et al. [15] used RT-PCR to amplify the RdPd gene from NV. In the present study, RV and NV gene amplification using qPCR was developed after selection of different open reading frame (ORF) fragments of the structural protein (VP) genes [9].

In this work, the SYBR GREEN technique was used to amplify a fragment of RdPd of norovirus and a fragment from the VP4 gene of rotavirus, which have been used to specifically detect norovirus and rotavirus genogroups [16,21]. We used the SYBR GREEN technique to detect and quantify our DNA product, because it is a simple, inexpensive and effective technique to detect both viruses in water [3]. We applied a method using a selected conservation region, namely, open reading frame 1 (ORF 1, part of the RdPd gene) in the case of NV, while a segment of gene 4 that codes for the outer capsid protein VP4, with an expected size of 345 bp, was selected for the case of RV. These are helpful to detect genogroup and genotype, respectively [2,22]. The limit of detection was up to 10^2^ virions, which is above the limit of viral particles that could be infective for an exposed individual [17]. Using this technique, the PCR products were analyzed by dissociative curves and by establishing dissociative temperatures.

Our results confirmed that the methodology used is viable for RV and NV identification in water samples. In earlier studies, RV and NV detection in water samples has been well documented [23,24,25]. Félix et al. [24] reported that NV was found in 15 beach water samples from Mexico, whereas Di Bartolo et al. [25] reported that RV was found in five water samples from Italy.

NV was detected during June (drought conditions) in the highest temperatures presented over the year, and during October, which was related to the rainy conditions in Bassaseachic National Park during 2013. Studies examining river water [26] and sewage water [27] have shown that there is a higher detection in the coldest months than in any other season, because viruses are more stable during December, when the daylight is shorter and the water is cooler [28]. Other studies showed that NV is frequently present in river water, treated wastewater and drinking water [2]. In comparison, Vieira et al. [29] reported NV GII concentration was close to zero at all sampling points in the Negro River Basin in Brazil, whereas Pérez-Sautu et al. [26] indicated a relationship of viral prevalence with the climate. As a result of the stability of the enteric viruses in water, most of the clinical cases have been reported in this cold season [24,30]. Our results differ from other studies that detected NV during the coldest months of the year; this could be due to contaminated water and represents a potential risk factor for regional diarrheic outbreaks.

The results showed seasonal differences in the presence/absence and concentration of viruses throughout the year in Bassaseachic Falls National Park. This type of human enteric virus could propagate in the human enteric tract and spread through human feces into water environments [21]. Over the seasons, RV and NV reported lower copies/L than other studies previously reported in Costa Rica in domestic wastewater [31]. Rohayem [32] and Japhet et al. [33] showed that climate changes in the temperature, precipitation, wind and even humidity can generate a suitable environment for enteric viruses to survive in. Kaas et al. [34] reported a weak association between the environment and the presence of NV. In addition, the silent and asymptomatic transmission of NV among village habitants due to open defecation may favor the NV prevalence in water [32,35,36,37,38].

RV is related to wastewater discharges. In this study, RV was detected during March and June. Our results are comparable with those reported by Fongaro et al. [6] in Florianopolis, Santa Catarina, Brazil, which showed 95–350 copies/reaction. In other studies, authors reported a higher detection during the coldest months, with most infection cases also occurring during the winter season [11,39]. In contrast, Fongaro et al. [28] detected up to 65% positive samples in all seasons, which indicated RV prevalence during the year, although this was found to be higher in the winter season. Assis et al. [40] detected 54.1% of RV in winter and spring seasons as well as 70.8% during the summer, thus indicating that the transmission of these enteric viruses resulted in higher infection rates through the winter and spring seasons, as stated by the Center for Disease Control and Prevention [41]. Patel et al. [42] indicated that transmission patterns, host behavior and susceptibility may contribute to the seasonal prevalence of RV, along with climate conditions. In Mexico, the Consejo Nacional de Agua (CONAGUA) reported in 2013 a low range of precipitation (0.5 to 52 mm) in addition to a temperature range of 18 to 27 °C for the summer season. This indicates that these environmental conditions could be permissive for RV prevalence. Therefore, we need more annual and environmental studies to help understand this relationship.

NV and RV identification, based on phylogenetic analyses, confirmed that both viruses had human origin, implying that contamination came from a human source. This may be due to open defecation from inhabitants around Bassaseachic Falls National Park. This may foster further investigation in this area to demonstrate that the quantity and prevalence of the virus could cause diarrheic outbreaks, in addition to providing new strategies in order to prevent them. Future work will focus on the detection of enteric viruses in contaminated sources and the examination of environmental conditions that allow for the prevalence of these viruses.

## 5. Conclusions

We confirmed the presence and prevalence of human rotavirus and norovirus in the surface water collected at Bassaseachic Falls National Park (west of Chihuahua, Mexico). These findings serve as a reminder for the need of surveillance to protect inhabitants during seasons when the environmental conditions are more permissive for rotavirus and norovirus prevalence, in order to reduce diarrhea outbreaks.

## Figures and Tables

**Table 1:** Oligonucleotides used for rotavirus detection.

Virus	Primer	5′–3′ Sequence		Amplicon Size	Reference
Rotavirus	CON 3	TGGCTTCGCCATTTTATAGACA	Forward	345 bp	Gentsch et al. [14]
Rotavirus	IT-I	TCTACTTGGATAACGTGC	Reverse		

**Table 2:** Oligonucleotides used for norovirus detection.

Virus	Primer	5´–3´ Sequence		Amplicon size	Reference
Norovirus	JV12	ATACCACTATGATGCAGATTA	Forward	326 bp	Thorven et al. [15]
Norovirus	JV13	TCATCATCACCATAGAAAGAG	Reverse		

**Table 3:** Detection of RNA from viruses in environmental water samples in different seasons.

Season	Volume (L)	Norovirus Copies/L	Rotavirus Copies/L
March	12	-	9.8 × 10^1^
June	11	1.0 × 10^2^	1.8 × 10^2^
October	13	5.27 × 10^2^	-
December	13	-	-

## Supplementary Materials

The following are available online at www.mdpi.com/1660-4601/14/5/482/s1, Table S1: Sample site from the Bassaseachic Falls National Park, Table S2: Reverse Transcrption conditions to detect Rotavirus, Table S3: Polimerase chaine reaction conditions to detect RV, Table S4: RT conditions to detect Norovirus, Table S5: PCR conditions to detect Norovirus, Table S6: qPCR conditions to detect Rotavirus, Table S7: qPCR conditions to detect Norovirus, Figure S1: Standard curve of the norovirus RdPd gene. (A) Lineal equation with a slope −3.229; (B) Products dissociation curve, Figure S2: Standard curve of the four genes from rotavirus. (A) Lineal equation with a slope −3.332; (B) Products dissociation curve.

## Abbreviations

RT-PCR, Reverse Transcriptase Polymerase Chain Reaction; CONANP, Comisión Nacional de Áreas Naturales Protegidas; qPCR, Real time Polymerase Chain Reaction; NV, norovirus; RV, rotavirus; CDC, Center for Disease Control and Prevention; CONAGUA, Comisión Nacional del Agua; VIRADEL, Virus absorption and elution technique.
