# Supplementary material for: Prevalence of Rotavirus Genogroup A and Norovirus Genogroup II in Bassaseachic Falls National Park Surface Waters in Chihuahua, Mexico
Source: Int J Environ Res Public Health. 2017 May 5;14(5):482. doi: 10.3390/ijerph14050482 (PMC5451933; doi:10.3390/ijerph14050482)
Supplement: Supplementary file 1 [file ijerph-14-00482-s001.pdf]

Supplementary Materials: Prevalence of Rotavirus Genogroup A and Norovirus Genogroup II in Bassaseachic Falls National Park Surface Waters in Chihuahua, Mexico

Ma. Carmen E. Delgado-Gardea, Patricia Tamez-Guerra, Ricardo Gomez-Flores, Aurora Mendieta-Mendoza, Francisco Javier Zavala-Díaz de la Serna, Juan Francisco Contreras-Cordero, Gilberto Erosa-de la Vega, María Concepción Pérez-Recoder, Blanca Sánchez-Ramírez, Carmen González-Horta and Rocío Infante-Ramírez

Table S1. Sample site from the Bassaseachic Falls National Park.

Sampling ID and Location		Geographical Coordinates		
**Number**	**Sample site**	**MSL ***	**NORTH**	**WEST**
1	Pozo Cascada de Basaseachi		28°07′59″	−108°15′00″
2	Cascada del Plan		28°07′59″	−108°15′00″
3	Río El Durazno	1,952	28°10′806″	−108°12′855″
4	Río Basaseachi	1,952	28°10′804″	−108°12′687″
5	Y. Punto de unión del Río Basaseachi y Rio el Durazno.	1,952	28°10′802″	−108°12′752″
6	Arroyo de Baquiriachi	2,022	28°12′680″	−108°13′341″
7	Aguaje de Basaseachi	2,052	28°13′992″	−108°12′453″
8	Arroyo de Las Estrellas	2,010	28°11′500″	−108°10′444″
9	Grifo CONANP	1,976	28°09′803″	−108°12′773″
10	Arroyo de Betorachi	1,932	28°10′928″	−108°11′067″
11	Arroyo de la laguna de oxidación	1,996	28°11′837″	−108°12′809″
12	Cahuisori	1,976	28°12′680″	−108°13′341″
13	Mirador de la Cascada		28°07′59″	−108°15′00″

* MSL: Meters above sea level

Table S2. Reverse Transcrption conditions to detect Rotavirus.

	**Reagent**	**[Initial]**	**[Final]**	**Volume**
**Step 1.**	RNA	50 ng	23.8 ng	2 μL
	Con 2 (Oligo T4)	1 μm	0.23 μm	1 μL
	H_2O injectable (PISA)	-	-	1.2 μL
			**Final volume =**	4.2 μL
	Thermocycler program: 1 cycle at 94ºC for 5 minutes.			
**Step 2.**	RT Buffer (Promega)	5X	1 X	2 μL
	$MgCl_2$ (Promega)	50 mM	2.5 mM	0.5 μL
	dNTP's (Promega)	2.5 mM	0.8 mM	3.2 μL
	RT enzyme (Promega)	200 U/μL	20 U	0.1 μL
			**Final volume =**	4.2 μL

Thermocycler program: 1 cycle at 94 °C for 30 min.

Table S3. Polimerase chaine reaction conditions to detect RV.

Reagent	[Initial]	[Final]	Volume
H_2O injectable (PISA)	-	-	16.3 μL
Buffer PCR (Promega)	10X	1X	2.5 μL
$MgCl_2$ (Promega)	50 mM	2 mM	1 μL
Con 3 (Oligo T4)	25 μM	1 μM	1 μL
Con 2 (Oligo T4)	25 μM	1 μM	1 μL
dNTP's (Promega)	2.5 mM	0.4 mM	2 μL
Taq polymerase (Promega)	5 U/μL	0.04 U	0.2 μL
cDNA	-	-	1 μL
		**Final volume =**	25 μL

Thermocycler program: 1 cycle at 94 °C for 90 s; 30 cycles at 94 °C for 30 s, 42 °C for 30 s and 72 °C for 40 s; and 1 final cycle at 72 °C for 7 min.

Table S4. RT conditions to detect Norovirus.

Reagent	[Initial]	[Final]	Volume
RNA	50 ng	12.5 ng	5 μL
JV13 (Oligo T4)	12 μm	2.5 μm	4 μL
H_2O injectable (PISA)	-	-	6.8 μL
RT Buffer (Promega)	5X	0.5 X	2 μL
$MgCl_2$ (Promega)	50 mM	3 mM	1.2 μL
dNTP's (Promega)	25 mM	1 mM	0.8 μL
RT enzyme (Promega)	200 U/μL	2 U	0.2 μL
	**Final Volume =**		20 μL

Thermocycler program: 1 cycle at 42 °C for 60 min.

Table S5. PCR conditions to detect Norovirus.

Reagent	[Initial]	[Final]	Volume
H_2O injectable (PISA)	-	-	8.2 μL
PCR Buffer (Promega)	5X	1X	4 μL
$MgCl_2$ (Promega)	25 mM	1.5 mM	1.2 μL
JV12 (Oligo T4)	12 μM	0.75 μM	1.24 μL
dNTP's (Promega)	25 mM	0.2 mM	0.16 μL
Taq polymerase (Promega)	5 U/μL	0.04 U	0.2 μL
cDNA	-	-	5 μL
	**Final Volume =**		20 μL

Thermocycler program: 1 cycle at 94 °C for 60 s; 40 cycles at 94 °C for 60 s, 37 °C for 30 s and 74 °C for 60 s; and 1 final cycle at 74 °C for 7 min.

Table S6. qPCR conditions to detect Rotavirus.

Reagent	[Initial]	[Final]	Volume
qPCR Buffer	2X	1X	10μL
DYE ROX 30 nm	-	-	0.3 μL
IT-I/Con 2 (Fw) (Oligo T4)	4 μM	0.5 μM	2.5 μL
CON3 (Rw) (Oligo T4)	4 μM	0.5 μM	2.5 μL
Plasmidic DNA	-	-	1μL
H_2O injectable (PISA)	-	-	3.7
	**Final Volume =**		20 μL

Thermocycler program: 1 cycle at 95 °C for 3 min; 45 cycles at 95 °C for 30 s, 53 °C for 20 s and 72 °C for 30 s. Thermocycler program for dissociation curve: 1 cycle at 95 °C for 60 s, 53ºC for 30 s and 95 °C for 30 s.

Table S7. qPCR conditions to detect Norovirus.

Reagent	[Initial]	[Final]	Volume
Buffer qPCR	2X	1X	10μL
DYE ROX 30 nm	-	-	0.3 μL
JV12 (Fw) (Oligo T4, Irapuato, México)	4 μM	0.2 μM	1 μL
JV13 (Rw) (Oligo T4, Irapuato, México)	4 μM	0.2 μM	1 μL
Plasmidic DNA	-	-	1 μL
H_2O inyectable (PISA, México)	-	-	6.7
		**Final Volume =**	20 μL

Thermocycler program: 1 cycle at 95 °C for 3 min; 40 cycles at 95 °C for 10 s , 48 °C for 20 s and 60 °C for 42 s . Thermocycler program for dissociation curve: 1 cycle at 95 °C for 60 s, 48 °C for 30 s and 95 °C for 30 s.

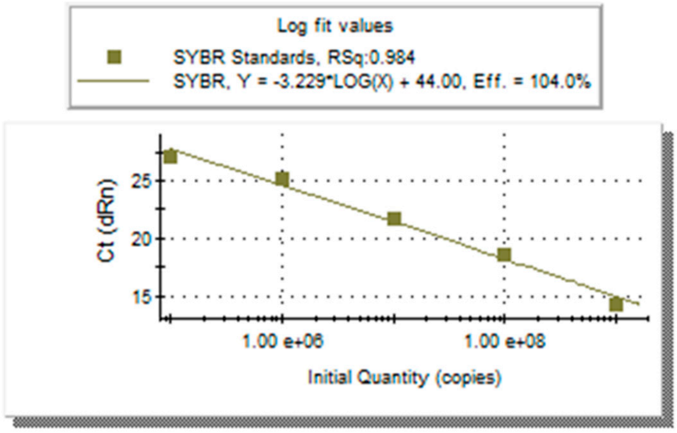

(A)

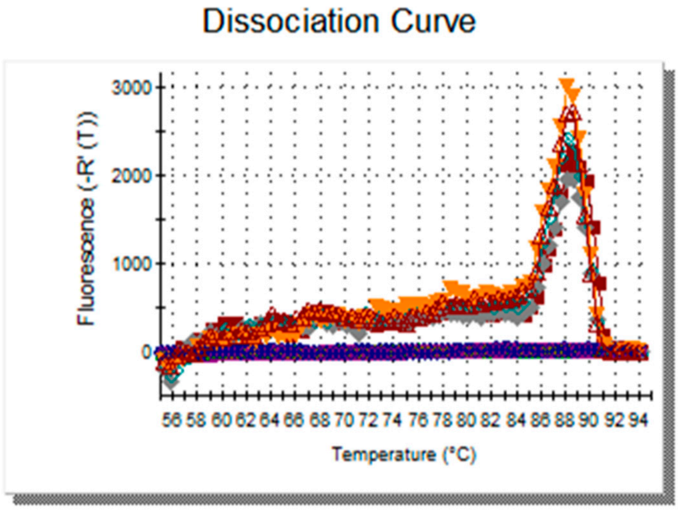

(B)

Figure S1. Standard curve of the norovirus RdPd gene. (A) Lineal equation with a slope −3.229; (B) Products dissociation curve.

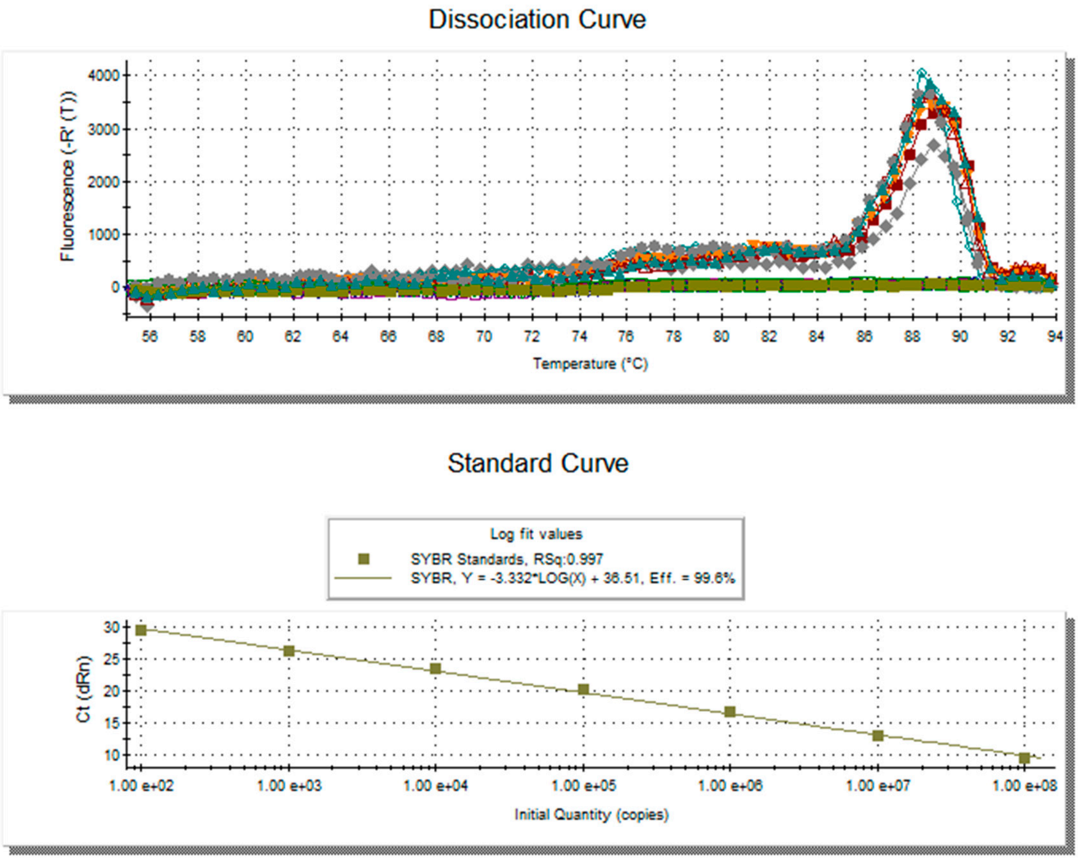

Figure S2. Standard curve of the four genes from rotavirus. (A) Lineal equation with a slope −3.332; (B) Products dissociation curve.

© 2017 by the authors; licensee MDPI, Basel, Switzerland. This article is an open access article distributed under the terms and conditions of the Creative Commons by Attribution (CC-BY) license (http://creativecommons.org/licenses/by/4.0/).
